# Patterns of psychotropic medicine use in pregnancy in the United States from 2006 to 2011 among women with private insurance

**DOI:** 10.1186/1471-2393-14-242

**Published:** 2014-07-22

**Authors:** Gillian E Hanley, Barbara Mintzes

**Affiliations:** 1 School of Population and Public Health, University of British Columbia, Vancouver, BC, Canada; 2 Child & Family Research Institute, Vancouver, BC, Canada; 3 Therapeutics Initiative, University of British Columbia, #307, 2176 Health Sciences Mall, Vancouver, BC V6T 1Z3, Canada

**Keywords:** Pregnancy, Prescription drugs, Psychiatric conditions in pregnancy, Psychotropic medicines, Administrative data, Depression, Anxiety

## Abstract

**Background:**

Psychiatric disorders are equally common during pregnancy as among non-pregnant women, and many of these conditions are treated with psychotropic medicines. Relatively little is known about patterns of use of many these agents during pregnancy, and specifically of how rates may have shifted during the last decade. We aimed to quantify the rate of pregnancy related exposures to categories of psychotropic medicines stratified according to the primary indication for use (antidepressants, antipsychotics, anxiolytics, and psychostimulants), trimester of pregnancy, trends over time and region, and indication for use.

**Methods:**

We conducted a retrospective cohort study of pregnancies among women in the Truven Health MarketScan database (source population 70 million Americans), which captures person-specific clinical use and includes detailed information on filled prescriptions, hospitalizations and outpatient visits for all privately insured employees and their dependents. We classified psychotropic medicines of interest using ATC level 3 accordingly: antipsychotics (N05A); anxiolytics (N05B); antidepressants (N06A); psychostimulants, agents used for ADHD and cognitive enhancement (N06B). We also examined temporal and regional trends in use.

**Results:**

We included 343,299 women who had a live birth between Jan 1, 2006 and Dec 31, 2011, of whom 10.3% were dispensed one or more psychotropic medicines during pregnancy. This rate varied from 6% to 15% between states. The rate of use of psychotropic medicines was relatively stable between 2006 and 2011. The most commonly used psychotropic medicines were selective serotonin reuptake inhibitors (5.1%) and benzodiazepine or benzodiazepine-like medicines (3.9%). Among psychotropic users, the most commonly associated psychiatric diagnosis was depression (25.0%), followed by anxiety disorders (24.4%). Approximately 1.6% of women used more than one category of psychotropic medicine in pregnancy, most commonly an antidepressant and an anxiolytic medicine (1.2%).

**Conclusions:**

Given this relatively high rate of use, the lack of evidence that the most frequently used medications improve birth outcomes and the safety concerns associated with both early and late pregnancy use for many frequently-used medications, there is a need for further study of factors driving psychotropic medication use during pregnancy.

## Background

In North America, there is evidence that use of psychotropic medicines during pregnancy, especially antidepressants, became increasingly prevalent in the early 2000’s [1]. Pregnancy was historically considered protective against mental health disturbances. More recent commentaries highlight pregnancy as a high-risk period [2]. Neither appears to be the case, with the most reliable estimates suggesting no significant difference in the rates of the most commonly diagnosed psychiatric disorders between pregnant and non-pregnant women of childbearing age, after adjustment for ethnicity, social class, age, health status and stressful life events [3-5]. The principal psychiatric disorders encountered during pregnancy include major depressive disorder, anxiety disorders, bipolar affective disorder, and schizophrenia [6]. These conditions are often treated with psychotropic medicines, and little is known about patterns of use of many these agents during pregnancy, and specifically of how rates may have shifted during the last decade.

While patterns of use during pregnancy of some categories of psychotropic medicines, most notably selective serotonin reuptake inhibitor (SSRI) antidepressants, have been well studied, estimates of the rate of use vary widely. For example, studies from the Nordic countries have reported that fewer than 2% of women use antidepressants during pregnancy [7], whereas one study in a Medicaid population in Tennessee reported use in 13.4% of all pregnancies [1]. Rates of use have also changed considerably over time [8-10]. This suggests that both temporal and geographic factors are important in understanding use of psychotropic medicines during pregnancy. These differences may reflect a range of factors, including underlying prevalence, rates of diagnosis and care seeking, and proportions with drug versus non-drug treatment.

The way that psychotropic medicines are prescribed has also shifted over time. For example, use of antipsychotics has increased, and the approved indications for antipsychotic drugs have expanded beyond psychotic disorders to include bipolar, major depressive, and anxiety disorders [11]. Previous research also suggests that the rate of antipsychotic use during pregnancy has been increasing [12]. ADHD is increasingly being diagnosed among adults as well as children [13]. Diagnosis and treatment of more than one mental health disorder at a time is not uncommon, and depression and anxiety disorders are often diagnosed as comorbid conditions [14-16].

A common methodological limitation to all studies examining outcomes of prescription drug use during pregnancy is confounding by the underlying indication for the medicine use and other systematic differences at baseline affecting both treatment decisions and outcomes. This is likely to be especially important for women who have multiple mental health disorders, as their outcomes may be confounded by their comorbid condition, and they may be exposed to more than one psychotropic medication. Thus, examining the use of one category of psychotropic medicine during pregnancy is unlikely to present a complete picture of the pharmacologic exposure of the infant or the mental health of the pregnant woman. Risks to the infant may also be higher with exposure to more than one class of psychotropic medicine. Oberlander et al. found higher risks of congenital heart defects among infants whose mothers were using both SSRI antidepressants and benzodiazepines during pregnancy [9]. Thus, understanding the complete picture of exposures to psychotropic medicines during pregnancy is important.

In this study we aim to quantify the rate of pregnancy related exposures to categories of psychotropic medicines stratified according to the primary indication for use (antidepressants, antipsychotics, anxiolytics, and psychostimulants), trimester of pregnancy, trends over time and region, and indication for use. We are especially interested in the proportion of women using different classes of drugs who initiate use during pregnancy, versus ongoing exposures among established users. We also examined the use of medicines from more than one category during pregnancy (e.g. an antidepressant and an anxiolytic). Defining how commonly psychotropic medicines are used during pregnancy, which psychotropic medicines are most often prescribed, what conditions are most commonly treated with psychotropic medicines, and how psychotropic conditions are managed during pregnancy will help define new research priorities in this field.

## Methods

We conducted a retrospective cohort study of pregnancies among women in the Truven Health MarketScan database (source population 70 million), which captures person-specific clinical use and includes detailed information on filled prescriptions, hospitalizations and outpatient visits for all insured employees and their dependents. These data come from a selection of large employers, health plans and government and public organizations (approximately 100 payers) across multiple states in the United States. Ethics approval was obtained from the Behavioral Research Ethics Board at the University of British Columbia.

### Study cohort

We included data from the first pregnancy for each mother that ended in a live birth in hospital between Jan 1, 2006 and Dec 31, 2011. Our data set includes primiparous and multiparous women, the latter with live births prior to 2006. Pregnancies were captured in the inpatient and outpatient data using ICD-9 diagnostic codes indicating delivery in the maternal hospital record and outpatient record. To ensure complete capture of prescription drugs used before and during pregnancy, we only included women who were enrolled continually during pregnancy and the three months pre-conception with no more than a one-month gap in their health insurance enrollment. As gestational age varied, the total number of months in which the women needed to be enrolled in the health insurance plan varied with it.

### Definitions of pregnancy periods

Maternal hospital records provided the date of admission for each in-hospital birth, but not the date of birth. We assumed that the date of admission was equal to the delivery date. The pregnancy period was built using the algorithm developed by Li et al., which was shown to correctly classify medication exposure status in most live born deliveries with a sensitivity and positive predictive value of ≥95% and a specificity and negative predictive value of almost 100% [17]. Using the gestational age (in days) calculated from the algorithm we estimated a date of conception by subtracting gestational days from the admission date. From this estimated conception date, we built several time periods for analysis of psychotropic drug exposures: 1) weeks 1 to 13 of gestation (first trimester); 2) Weeks 14 to 26 of gestation (second trimester), 3) week 27 of gestation to delivery (third trimester), 4) 6 months prior to conception (preconception) and, 5) six months postpartum (postpartum).

### Psychotropic drug use in pregnancy

Outpatient prescription drug claims include prescriptions dispensed to women at eligible pharmacies either through a mail-order or card program prescription drug claim, capturing all prescriptions dispensed that are covered by her insurance plan. We classified psychotropic medicines of interest using ATC level 3 accordingly: antipsychotics (N05A); anxiolytics (N05B); antidepressants (N06A); psychostimulants, agents used for ADHD and cognitive enhancement (N06B). Additional file 1 outlines all medicines in each category that were dispensed to a woman in our cohort during pregnancy. We excluded the caffeine and caffeine combinations that were included in N06B. We did not remove medicines that we suspected were being prescribed for non-psychological indications, such as the typical antipsychotics that appear to be used primarily as antiemetics, as these prescriptions still represent exposures to a psychotropic medicine from one of the relevant drug classes. We also present frequencies of women who filled prescriptions from more than 2 categories of psychotropic medicines during the perinatal period, and the use of 2 or more different antidepressant medicines during the perinatal period.

We defined exposure to a psychotropic medicine during pregnancy and according to trimester if a prescription was filled on a date within the relevant pregnancy period. To account for the possible overestimation that might result from including psychotropic medicines filled only once in the first trimester, we also present conservative numbers of exposures during pregnancy where we have removed women who filled only 1 prescription during the first trimester of pregnancy, as these might represent prescriptions filled prior to knowledge of the pregnancy and not used once the woman became aware she was pregnant.

### Patterns of prescription drug exposure

We defined prevalent users of psychotropics during pregnancy as women for whom the psychotropic was dispensed in the 6 months prior to pregnancy and then again in pregnancy. In contrast, incident users were those with no prescription for the psychotropic in the 6-months before pregnancy. Again we also present conservative estimates by removing the women who only filled one prescription for the psychotropic of interest in the first trimester from both groups. Finally we present incident use of psychotropic medicines in the postpartum period, defined as initiation of use during the 180 days following delivery. These women had no prescription for the psychotropic agent before or during pregnancy.

### Description of associated conditions

Medical conditions associated with use of psychotropics were identified using the *International Classification of Diseases, Ninth Revision* diagnosis codes in the patient’s hospital and outpatient records between 180 days before our estimated conception date and delivery. Additional file 2: Table S1 outlines the diagnostic codes used. The frequency of these conditions was determined in (1) all patients exposed to psychotropics agents during pregnancy (2) patients exposed to each category of psychotropic medicine (3) according to incident or prevalent use of psychotropic medicines and for the two most commonly used categories of psychotropic medicines, antidepressants and anxiolytics and (4) among women who did not fill prescriptions for psychotropic medicines in pregnancy.

### Temporal and regional trends in psychotropic medicine use during pregnancy

We examined the trends in exposure to any psychotropic medicine during pregnancy and to each category of psychotropic medicines annually from 2007 to 2011 according to the year of delivery. We examined regional variations in psychotropic prescribing by examining frequency of exposure to psychotropic medicines, antidepressants and anxiolytics by state.

### Statistical analyses

All analyses were descriptive of this commercially insured population. The results are parameters for this particular population rather than estimates based on a sample and thus we present the results without confidence intervals. All analyses were performed using either Stata version 13.0 (College Station, TX) or SAS version 9.3 (SAS Institute, Cary, NC).

## Results

There were 343,299 live births between Jan 1, 2006 and Dec 31, 2011 that met our enrollment criteria (not missing more than one month of enrollment for the 3 months prior to conception and during the pregnancy). The mean age of the women at the time of delivery was 30.3 years with a standard deviation of 5.6 years (median 30 and interquartile range 27 to 34); 4,895 (1.4%) pregnancies were multiple gestations, 29,733 (8.7%) were preterm deliveries. Overall 35,303 women (10.3%) were prescribed a psychotropic medicine during pregnancy (this number drops to 6.8% when we remove women who only filled one prescription during the first trimester of pregnancy). 24,776 (7.2%) women filled a prescription for a psychotropic medicine during the first trimester, 15,883 (4.6%) during the second trimester and 18,161 (5.3%) during the third trimester. The most common category of psychotropic medicine use during pregnancy was antidepressants, with 22,275 (6.5%) women filling an antidepressant during pregnancy (conservative estimates are 15,097 (4.4%)), followed by anxiolytics which were filled by 14,535 (4.2%) of pregnant women in our cohort (conservatives estimates are 9,235 (2.7%)). Antipsychotics and stimulants were used during pregnancy by 2,373 (1.1%) and 2,062 (0.6%) respectively (Table 1). There were 5,423 (1.6%) women using more than one category of psychotropic medicine during pregnancy, and most of these women were using a combination of antidepressants and anxiolytics (4,068, 1.2%).

**Table 1:** Summary measures of psychotropic prescription drug use before, during and after pregnancy

**N = 343299**	**6 months preconcept n (%)**	**1st trimester n (%)**	**2nd trimester n (%)**	**3rd trimester n (%)**	**Pregnancy total n (%)**	**Pregnancy total with >1 Rx n (%)**
Any psychotropic	33,995 (9.9)	24776 (7.2)	15883 (4.6)	18161 (5.3)	35303 (10.3)	23261 (6.8)
Antidepressant	23,083 (6.7)	17214 (5.0)	12235 (3.6)	11937 (3.5)	22275 (6.5)	15097 (4.4)
SSRI	16524 (4.8)	12881 (3.8)	9773 (2.9)	9751 (2.8)	17410 (5.1)	12278 (3.6)
SNRI	3170 (0.9)	2175 (0.6)	973 (0.3)	805 (0.2)	2382 (0.8)	1075 (0.3)
Tricyclic	1222 (0.4)	611 (0.2)	236 (0.07)	209 (0.06)	784 (0.2)	308 (0.1)
Other	4390 (1.3)	3085 (0.9)	1875 (0.6)	1634 (0.5)	4019 (1.2)	1026 (0.3)
≥ 2 antidepressants	2776 (0.8)	2132 (0.6)	889 (0.3)	676 (0.2)	3521 (1.0)	1613 (0.5)
Anxiolytic	12969 (3.8)	7503 (2.2)	3881 (1.1)	7002 (2.0)	14535 (4.2)	9235 (2.7)
Benzo or benzo-like*	12231 (3.5)	6840 (2.0)	3391 (1.0)	6686 (2.0)	13486 (3.9)	8613 (2.5)
Other	950 (0.3)	809 (0.2)	549 (0.2)	377 (0.1)	1375 (0.4)	757 (0.2)
Antipsychotic	1344 (0.4)	1728 (0.5)	808 (0.2)	503 (0.1)	2373 (1.1)	1015 (0.3)
Atypical	899 (0.3)	604 (0.2)	315 (0.1)	301 (0.1)	742 (0.2)	388 (0.1)
Typical	357 (0.1)	1072 (0.3)	464 (0.1)	160 (0.05)	1573 (0.5)	995 (0.3)
Stimulant/ADHD	2798 (0.8)	1982 (0.6)	498 (0.1)	325 (0.1)	2062 (0.6)	545 (0.2)
≥ 2 categories of psychotropic**	5423 (1.6)	3339 (1.0)	1431 (0.4)	1519 (0.5)	5423 (1.6)	2390 (0.7)
Antidepressant + anxiolytic	4195 (1.2)	2293 (0.7)	1064 (0.3)	1247 (0.36)	4068 (1.2)	1630 (0.5)

*Includes benzodiazepines as well as eszopliclone, zolpiedem, and zaleplon.

**defined as prescriptions for ≥ 1 class of psychotropic medicine during pregnancy e.g. a benzodiazepine and an antidepressant; does not include women who used ≥ 1 medicine within the same class (e.g. two antidepressants).

### Patterns of prescription drug exposure

Rates of use of all psychotropic medicines were higher in the 6 months preconception than in the first trimester and decreased further in the second trimester, likely reflecting women who stopped medicines after becoming aware of their pregnancy. This pattern was consistent across all categories of psychotropic medicine except for typical antipsychotics, which were used considerably more in the first trimester of pregnancy, likely as an antiemetic.

The most common class of antidepressants dispensed during pregnancy were SSRIs which were dispensed to 17,410 (5.1%) of women followed by selective norepinephrine reuptake inhibitors (dispensed to 2,382 or 0.8% of women). Table 2 lists the most common psychotropic medicines among each category and includes all medicines that were used by at least 0.1% of the study population. Among SSRIs the most commonly dispensed medicines were sertraline, fluoxetine, and escitalopram with 8,432 (2.5%), 3,605 (1.1%), and 3,481 (1.0%) of women filling a prescription for these medicines during pregnancy respectively. Nearly all of the anxiolytics prescribed were benzodiazepines or benzodiazepine-like medicines (n = 13,486, 3.9%), the latter including zaleplon, zolpidem, and eszopiclone. The most common benzodiazepine or benzodiazepine-like medicines prescribed were zolpidem (n = 8,239, 2.4%), alprazolam (n = 2,654, 0.8%), diazepam (n = 1,884, 0.6%) and lorazepam (n = 1,233, 0.4%).

**Table 2:** Most common psychotropic exposures (not exhaustive of all drug exposures—only medicines that were used by ≥ 0.1% of the study population)

**Drug class**	**Number exposed during pregnancy**	Conservative number exposed during pregnancy*	**Number who filled more than 1 Rx during pregnancy**
SSRI	**17414 (5.1)**	**13379 (3.9)**	**11142 (3.3)**
Sertraline	8432 (2.5)	6936 (2.0)	5360 (1.6)
Fluoxetine	3605 (1.1)	2694 (0.8)	2300 (0.7)
Escitalopram	3481 (1.0)	2310 (0.7)	1951 (0.6)
Citalopram	2295 (0.7)	2310 (0.7)	1246 (0.4)
Benzodiazepines (and benzo like)	**13486 (3.9)**	**9399 (2.7)**	**4304 (1.3)**
Zolpidem	8239 (2.4)	7238 (2.1)	2676 (0.8)
Alprazolam	2654 (0.8)	1288 (0.4)	893 (0.3)
Diazepam	1884 (0.6)	384 (0.1)	178 (0.05)
Lorazepam	1233 (0.4)	634 (0.2)	302 (0.1)
Other antidepressants	**4019 (1.2)**	**2658 (0.8)**	**2164 (0.6)**
Bupropion	3399 (1.0)	2351 (0.7)	1925 (0.6)
Trazodone	602 (0.2)	270 (0.1)	189 (0.1)
SNRIs	**2382 (0.7)**	**1474 (0.4)**	**1377 (0.4)**
Venlafaxine	1398 (0.4)	936 (0.3)	875 (0.3)
Duloxetine	795 (0.2)	449 (0.1)	416 (0.1)
Desvenlafaxine	210 (0.1)	100 (0.03)	96 (0.03)
Typical antipsychotics	**1573 (0.5)**	**712 (0.2)**	**274 (0.1)**
Prochlorperazine	1527 (0.4)	682 (0.2)	250 (0.1)
Other anxiolytics	**1375 (0.4)**	**820 (0.2)**	**375 (0.1)**
Buspirone	745 (0.2)	520 (0.2)	288 (0.1)
Scopolamine	535 (0.2)	248 (0.1)	56 (0.02)
Amphetamine	**1291 (0.4)**	**657 (0.2)**	**633 (0.2)**
Cyclic antidepressants	**784 (0.2)**	**385 (0.1)**	**272 (0.1)**
Amitriptyline	490 (0.1)	239 (0.1)	162 (0.05)
Nortriptyline	173 (0.1)	75 (0.02)	56 (0.02)
Atypical antipsychotics	**742 (0.2)**	**465 (0.1)**	**397 (0.1)**
Quetiapine	363 (0.1)	228 (0.1)	191 (0.06)

*Removes women who only filled one prescription in the first trimester.

The majority of typical antipsychotic prescriptions during pregnancy were for prochloperazine (n = 1527, 0.4%), and nearly half of these prescriptions occurred only during the first trimester. The conservative number of women exposed, excluding women with a single prescription in the first trimester, drops to 682 (0.2%). If prochloperazine was primarily being used as an antiemetic, women may have filled a single prescription for short-term relief of first trimester nausea. As we could not distinguish between this situation and women who discontinued drug use when they knew they were pregnant, we retained the same definition of conservative use as with other medications. Only 17% of women with prescriptions for typical antipsychotics during pregnancy refilled their prescription (n = 274, 0.1%). The most commonly used atypical antipsychotic was quetiapine, which has been approved to treat major depressive disorder. However, use of quetiapine in pregnancy was uncommon (n = 363, 0.1%).

Table 3 shows the mean, median and interquartile range for the cumulative numbers of days supply during the pregnancy among the patients dispensed each category of psychotropic medicine. The median duration of antidepressant use during pregnancy was 90 days with a mean duration of 125.3 days, reflecting exposure for nearly half the pregnancy. This duration of exposure was similar among women using SSRIs and slightly shorter among women using SNRIs (median 60 days, mean 111.1) and other antidepressants (median 60 days, mean 100.0 days). Anxiolytic exposure was much shorter with a median exposure of 30 days and a mean of 43.2 days. The shortest duration of exposure was among the typical antipsychotics, including prochlorperazine (97% of use in this class), amitriptyline, chlorpromazine, fluphenazine, haloperidol, loxapine, perphenazine, thioridazine, and trifluoperazine. Typical antipsychotics were used for a median of 8 days and a mean of 14.7 days. Stimulants had median exposure duration of 49 days (mean 79.8).

**Table 3:** For psychotropic medicines dispensed during pregnancy, number of days exposure during pregnancy by type

	**Median**	**25th percentile**	**75th percentile**	**Mean ± SD**
Any psychotropic	60	30	160	104 ± 118.2
Antidepressant	90	30	210	125.3 ± 103.2
SSRI	90	30	180	118.9 ± 91.4
SNRI	60	30	180	111.1 ± 98.6
Cyclic	30	30	80	69.1 ± 72.5
Other	60	30	150	100.0 ± 90.3
Anxiolytic	30	10	40	43.2 ± 65.6
Benzo or benzo-like	29	10	40	41.8 ± 64.0
Other	30	12	40	46.7 ± 59.7
Antipsychotic	14	7	30	46.4 ± 81.7
Atypical	60	30	150	103.8 ± 102.5
Typical	8	5	15	14.7 ± 24.4
Stimulant/ADHD	49	30	90	79.8 ± 89.9

Table 4 outlines psychotropic medicine use during pregnancy according to whether the use represents prevalent or incident use in pregnancy. Most use of antidepressants during pregnancy represents prevalent use (n = 15,253, 4.4%; Table 4). In contrast, anxiolytics (primarily benzodiazepines and benzodiazepine-like medicines) were most often initiated during pregnancy (n = 9,215, 2.7% incident users versus n = 4271, 1.2% prevalent users). Typical antipsychotics were also used primarily by incident users in pregnancy (Table 4).

**Table 4:** Prevalent and incident use of psychotropic medicines during the perinatal period

**N = 343,299**	**Prevalent use in pregnancy**	Prevalent use in pregnancy; > 1 Rx*	**Incident use in pregnancy**	Incident use in pregnancy > 1 Rx*	**Incident use postpartum**
Antidepressants	15253 (4.4)	11588 (3.4)	7,022 (2.1)	5353 (1.6)	15652 (4.6)
SSRI	10998 (3.2)	8300 (2.4)	6,416 (1.9)	5079 (1.5)	14625 (4.3)
Anxiolytics	4574 (1.3)	2992 (0.9)	9961 (2.9)	7100 (2.1)	7583 (2.2)
Benzo**	4271 (1.2)	2742 (0.8)	9,215 (2.7)	6657 (1.9)	7319 (2.1)
Antipsychotic	559 (0.2)	365 (0.1)	1814 (0.5)	854 (0.3)	773 (0.2)
Typical antipsychotic	27 (0.01)	14 (0.00)	1546 (0.5)	698 (0.2)	326 (0.1)
Atypical antipsychotic	480 (0.1)	307 (0.09)	262 (0.1)	158 (0.05)	447 (0.1)
ADHD	1741 (0.5)	886 (0.3)	321 (0.1)	135 (0.04)	604 (0.2)

*Removes women who only filled one prescription in the first trimester.

**benzodiazepine or benzodiazepine like product.

### Description of associated conditions

Table 5 shows the relevant mental health conditions in patients with and without psychotropic medicine use during pregnancy. Of the 35,303 psychotropic medicine users during pregnancy, just under half had a relevant diagnosis (49.7%) at any time during pregnancy or in the six months pre-conception. Of these, the most common diagnoses were depressive disorders (25.0%) and anxiety disorders (24.4%); 31.5% of antidepressant users had been diagnosed with anxiety compared to 35.3% diagnosed with a depressive disorder. Just under half of all users of stimulants had a diagnosis of ADHD (49.6%) and 72.3% of all stimulant users had a diagnosis of at least one mental health disorder. Women who were using more than one psychotropic during pregnancy were most likely to have at least one relevant psychiatric diagnosis (73.9%). There were 11,181 women with a diagnosis of depression and 14,102 women with a diagnosis of an anxiety disorder who did not use psychotropic medicines during pregnancy representing 57% and 62% of women in our data set with these diagnoses, respectively. Of the 11,217 women diagnosed with acute stress or adjustment disorder, 3176 (28%) were prescribed a psychotropic drug, most often an antidepressant.

**Table 5:** Diagnosed maternal conditions in the year prior to or during pregnancy, stratified by exposure to psychotropic medicines

**Diagnoses, n (%)**	**Any psychotropic N = 35303**	**Antidepressant N = 22275**	**Anxiolytic N = 14535**	**Stimulants for ADHD N = 2062**	**Antipsychotic N = 2373**	**No psycho-tropic use N = 307996**	**More than one N = 5423**
Bipolar disorder	1637 (4.6)	1225 (5.5)	581 (4.0)	499 (24.2)	98 (4.1)	1747 (0.6)	634 (11.7)
Major depressive disorder	8814 (25.0)	7874 (35.3)	2414 (16.6)	488 (23.7)	218 (9.2)	11861 (3.9)	2036 (37.5)
Anxiety	8611 (24.4)	7006 (31.5)	3188 (21.9)	487 (23.6)	213 (9.0)	14102 (4.6)	2127 (39.2)
Adj/Acute stress^‡^	3176 (9.0)	2335 (10.5)	1261 (8.7)	179 (8.7)	211 (8.9)	8041 (2.6)	725 (13.4)
Schizophrenia	48 (0.1)	28 (0.1)	11 (0.1)	35 (1.7)	1 (0.04)	34 (0.01)	22 (0.4)
Personality disorder	143 (0.4)	116 (0.5)	40 (0.3)	27 (1.3)	3 (0.1)	141 (0.05)	38 (0.7)
Sleep disorder	1799 (5.1)	1130 (5.1)	1033 (7.1)	75 (3.6)	34 (1.4)	3255 (1.1)	472 (8.7)
ADHD	1347 (3.8)	546 (2.5)	292 (2.0)	1023 (49.6)	73 (3.1)	1119 (0.4)	469 (8.6)
At least one relevant diagnosis	17561 (49.7)	13655 (61.3)	5786 (39.8)	1490 (72.3)	1086 (45.8)	25707 (8.3)	4007 (73.9)

^‡^A diagnosis of adjustment reaction and/or acute stress.

Table 6 shows diagnoses for mental health disorders according to prevalent and incident use of psychotropic medicines, antidepressants and anxiolytics (as well as conservative prevalent and incident use). The rate of users with a relevant diagnosis is nearly double for prevalent users of psychotropic medicines compared with incident users (63.1% versus 31.1% respectively).

**Table 6:** Diagnoses by prevalent and incident use of psychotropics, antidepressants and anxiolytics

	**Prevalent use during pregnancy**	Conservative prevalent use*	**Incident use in pregnancy**	Conservative Incident use*
**Psychotropic users**	**N = 20481**	**N = 15689**	**N = 14822**	**N = 10798**
Bipolar disorder	1294 (6.3)	1105 (7.0)	342 (2.3)	278 (2.6)
MDD	6724 (32.8)	5680 (36.2)	2084 (14.1)	1730 (16.0)
Anxiety	6450 (31.5)	5266 (33.6)	2153 (14.5)	1651 (15.3)
Sleep disorder	1360 (6.6)	1076 (6.9)	439 (3.0)	319 (3.0)
ADHD	1208 (5.9)	877 (5.6)	139 (1.0)	86 (0.8)
Adj/acute^‡^	2098 (10.2)	1652 (10.5)	1078 (7.3)	800 (7.4)
Any relevant diagnosis	12921 (63.1)	10400 (66.3)	4640 (31.3)	3567 (33.0)
**Antidepressant users**	**N = 15253**	**N = 11588**	**N = 7022**	**N = 5353**
Bipolar disorder	895 (5.9)	721 (6.2)	329 (4.7)	250 (4.7)
MDD	5858 (38.4)	4789 (41.3)	2010 (28.6)	1629 (30.4)
Anxiety	5176 (33.9)	4118 (35.5)	1822 (26.0)	1372 (25.6)
Sleep disorder	844 (5.5)	637 (5.5)	286 (4.1)	210 (3.9)
ADHD	402 (2.6)	315 (2.7)	144 (2.1)	104 (1.9)
Adj/acute^‡^	1623 (10.6)	1241 (10.7)	712 (10.1)	532 (9.9)
Any relevant diagnosis	9967 (65.3)	7896 (68.1)	3688 (52.5)	2821 (52.7)
**Anxiolytic users**	**N = 4574**	**N = 2993**	**N = 9961**	**N = 7099**
Bipolar disorder	325 (7.1)	240 (8.0)	255 (2.6)	181 (2.6)
MDD	1181 (25.8)	826 (27.6)	1227 (12.3)	886 (12.5)
Anxiety	1609 (35.2)	1075 (35.9)	1571 (15.8)	1016 (14.3)
Sleep disorder	596 (13.0)	407 (13.6)	437 (4.4)	294 (4.1)
ADHD	166 (3.6)	119 (4.0)	126 (1.3)	102 (1.4)
Adj/acute^‡^	506 (11.1)	332 (11.1)	755 (7.6)	524 (7.4)
Any relevant diagnosis	2753 (60.2)	1858 (62.1)	3033 (30.5)	2080 (29.3)

*Removes women who filled 1 prescription in the first trimester.

^‡^A diagnosis of adjustment reaction and/or acute stress.

### Temporal and regional trends

**Figure 1:**
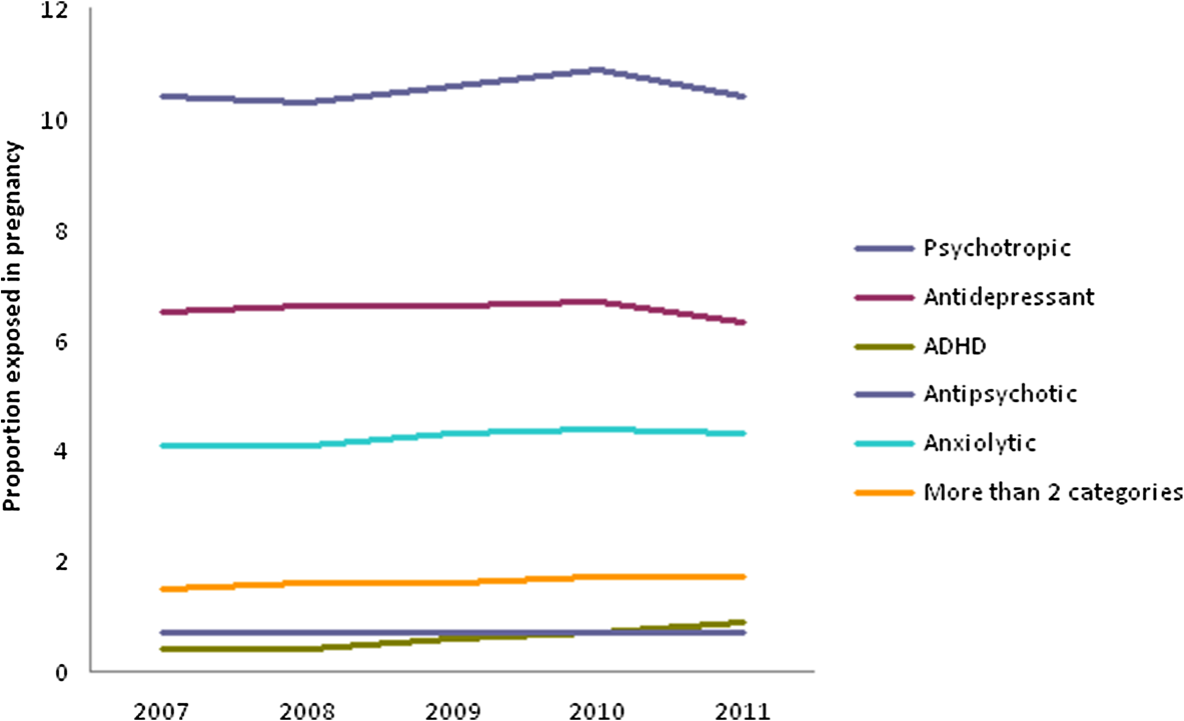
Psychotropic medicine use during pregnancy by year.

**Figure 2:**
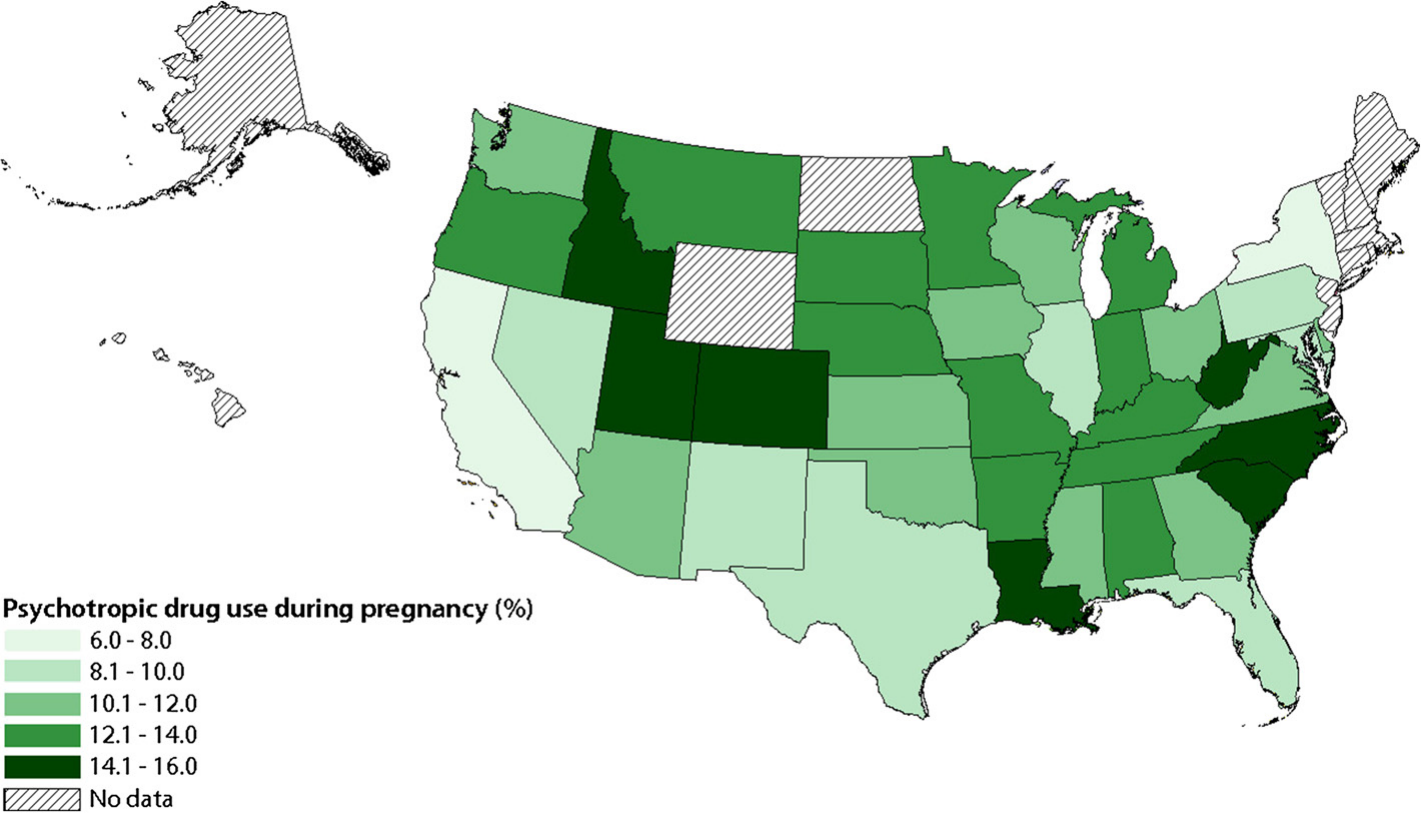
Psychotropic medicine use during pregnancy by state.

Figure 1 shows psychotropic medicine use during pregnancy across each year of study. The figure indicates remarkable consistency in exposure to psychotropic medicines during the study period. There was a slight decrease in antidepressant use between 2010 and 2011 from 6.7% to 6.4%, and the use of stimulants increased from 0.4% in 2007 to 0.9% in 2011. Figure 2 shows the rate of psychotropic exposure during pregnancy by state. Significant regional differences are observed with the lowest rates of psychotropic use being observed in New York (6.44%) and California (6.99%) and the highest rates of use in Idaho (15.41%), Louisiana (14.94%), Utah (14.80%), West Virginia (14.44%), and South Carolina (14.17%). A number of the Southern states had higher than average rates, with Alabama, Arkansas, Kentucky, Louisiana, North and South Carolina, Indiana, Tennessee and West Virginia all having rates above 12%.

## Discussion

In this analysis of nearly 350,000 pregnancies among women with private health insurance from across the United States, we found that at least one psychotropic medicine was dispensed to 1 in 10 pregnant women during pregnancy. The most commonly used medicines during pregnancy were antidepressants (selective serotonin reuptake inhibitors specifically) and anxiolytics (benzodiazepine and benzodiazepine-like medicines). We also report that 1.6% of women were using two or more different categories of psychotropic medicines during pregnancy, most often an antidepressant and an anxiolytic. The median duration of exposure was 60 days, ranging from 29 to 90 days for all categories of medications except for typical antipsychotics, which are often used as antiemetics. Relatively few women received atypical antipsychotics (1.1%) or stimulants or other drugs for ADHD (0.6%). We found very little variation in the rate of exposure over the study period, with approximately 10% of pregnant women using a psychotropic medicine during pregnancy in each year between 2007 and 2011.

The most common psychiatric diagnoses in this cohort in the period from six months pre-pregnancy to the end of pregnancy was anxiety (n = 22,713) followed by depression (n = 20,675), with 37.9% and 42.6% of those with each diagnosis, respectively, receiving psychotropic medications in pregnancy. The latter is similar to the 40.3% rate of antidepressant use among women with a relevant diagnosis in a national US study of over one million pregnant low-income women covered by Medicaid from 2000 to 2007 [18]. Among women taking antidepressants, anxiety diagnoses (31.5%) were nearly as prevalent as depression diagnoses (35.3%), and there were twice as many antidepressants users with an anxiety diagnosis than anxiolytic users with an anxiety diagnosis (7,006 compared with 3,176).

What do these findings mean for maternal and infant health and well-being? While the evidence in this area is complex and often contradictory, and a full evidence review is beyond the scope of this article, a few areas of concern merit discussion in order to provide necessary context for our results. Pregnant women with depression have worse birth outcomes, on average, than women without a psychiatric diagnosis, and this is often used as a rationale to support drug treatment in pregnancy [19]. However, the evidence on depression and pregnancy outcomes has been criticized for inadequately addressing confounding [20], including socio-economic factors [19,21], psychiatric diagnoses [22] and pregnancy complications [23,24], and it is often unclear whether depression and anxiety diagnoses precede or follow pregnancy complications [25]. More importantly, to date there is no evidence that antidepressants for depression or anxiolytics for anxiety mitigate poorer health outcomes among women with depression or anxiety-related diagnoses [26-28]. The medicines used to treat these mood disorders have been associated with poorer birth outcomes [29,30]. Selective serotonin use in pregnancy has been associated with a number of adverse health outcomes, including a higher rate of miscarriage [31], pre-term birth [32], congenital heart malformations with first trimester exposure [33], and persistent pulmonary hypertension of the newborn [34]. The extent to which poorer health outcomes with antidepressant exposure are a result of unmeasured confounding by the underlying maternal depression or anxiety remains an open question [9]. There is less inconsistency with respect to postnatal adaptation syndrome, a syndrome that generally presents as a transient combination of respiratory distress, jitteriness, abnormal tone, tremors, restlessness, convulsions, jaundice, rigidity, and hypoglycaemia [35-38], and occurs in approximately 1/3rd of newborns exposed to SSRIs in utero [39]. The evidence on safety of use of atypical antipsychotics is sparse but tends to suggest an increased risk of NICU admission among exposed infants; however, the population of antipsychotic users tends to have much higher rates of many adverse conditions, which likely confounds this association [40,41]. There is some evidence from case–control studies of an increased risk of cleft palate with benzodiazepine use in the first trimester [42], and a neonatal withdrawal syndrome following third trimester use [42,43]. A research study from Taiwan has suggested that zolpidem, the most frequently used anxiolytic in our cohort, is associated with poorer infant outcomes; however, unmeasured confounding is again a very real problem in this literature [44].

Our results were not consistent with earlier studies reporting a trend of increased use of psychotropic medicines during pregnancy. Cooper et al. examined a publicly insured American Medicaid population in Tennessee and reported that antidepressant use more than doubled between 1999 and 2003 from 5% to 13% [1]. A more recent national study of women covered by Medicaid from 2000 to 2007 found that 8.1% were dispensed antidepressants in pregnancy [18]. They also found that antidepressant use declined following an FDA warning in 2003 on increased risk for suicidality in children and adolescents [18]. Although largely irrelevant to use in pregnancy, this may have led to greater overall caution. The higher rate reported (8.1% versus our rate of 6.7%) may reflect greater exposure among women of lower socio-economic status; however, there is only one year of overlap in the two analyses, and although we found no difference in exposure rate from 2007 to 2011, an earlier shift in rate of drug use may have occurred. Our results regarding higher rates of use of psychotropic medicines in Southern states is consistent with regional variation in depression diagnoses among adults reported in US epidemiological surveys [45]. Our study also indicates higher than average rates in some Mid-Western and Western states, a finding that differs from disease surveillance surveys but should be interpreted with caution, as we have some small numbers in some of these states (e.g. Idaho n = 785; Kansas n = 2,833)

We found little use of atypical antipsychotics (0.2%). Other research has reported an increasing trend in off-label use of antipsychotics in the general US population, including, for example, prescribing of quetiapine for insomnia [11]. While we reported a high rate of use of typical antipsychotics in the first trimester, given the median prescription length (8 days) and frequent incident use in the first trimester, we expect that this represents use as antiemetics. Prochlorperazine, the most commonly prescribed typical antipsychotic in our cohort, is indicated in the U.S. for severe nausea and vomiting. However, the drug’s label warns against use in pregnancy, “except in cases of severe nausea and vomiting that are so serious and intractable that, in the judgment of the physician, drug intervention is required and potential benefits outweigh possible hazards” [46].

Our study is subject to some limitations. Our data are drawn from a large private insurance claims database that includes women from across the United States (44 states are represented). However, a large proportion of births in the US occur to women who are covered by Medicaid [47], and it is unclear whether our findings generalize to these women. The same applies to our state-specific prescribing patterns, which only reflect prescribing patterns of this commercially insured population. However, the general regional trends we present are consistent with previous studies of prescription drug use during pregnancy [1,48]. We were also unable to identify pregnancies that ended in spontaneous abortions, or therapeutic abortions, a population which may have higher exposure to psychotropic medicines [31]. Any bias created through these omissions is likely to be in the direction of underestimating exposure.

Our measure of exposure is based on pharmacy dispensing of medication, and does not directly measure medication use by the pregnant women. To account for this limitation, we provide conservative estimates that remove women who filled only one prescription in the first trimester, as these women seem most likely to have filled prescriptions that might not have been used. Finally, we had to impute last menstrual period based on claims for labor and delivery, which provide information on preterm birth. Although this approach has been shown to be valid [17], some degree of misclassification is likely. We are also limited in our ability to detect diagnosed mental health conditions. While ICD-9 codes are helpful in identifying conditions, they do not reflect a gold-standard diagnosis and a preferable measure would be a diagnostic interview undertaken by a trained health care provider. We also report that only half of our psychotropic medicine users had a relevant mental health diagnosis. While this may reflect some missing diagnostic information due to one-month lapses in coverage, it is also consistent with results from the US National Ambulatory Medical care surveys indicating the lack of psychiatric diagnosis noted at two-thirds of primary care consultations at which an antidepressant was prescribed [49]. The fact that many of these medicines are commonly prescribed for other indications may also be a relevant factor, as our rates of diagnoses were higher among women using categories of medicines with less off-label use (e.g. stimulants to treat ADHD).

## Conclusions

In summary, approximately 10% of privately-insured women in the United States are dispensed one or more psychotropic medicine during pregnancy, with important regional variation, around 6% to 15%. The most commonly used psychotropic medicines are selective serotonin reuptake inhibitors and benzodiazepine or benzodiazepine-like medicines. The most commonly associated psychiatric diagnosis was depression, followed by anxiety disorders, which were mainly treated with antidepressants. Approximately 1.2% of women use both an antidepressant and an anxiolytic medicine during their pregnancy. Given these relatively high rates of use, the lack of evidence that the most frequently used medications improve birth outcomes, and the safety concerns raised both by early and late pregnancy use for many frequently-used medications, there is a need for further study of factors driving use of these medicines during pregnancy. For depression and anxiety disorders, alternative treatment options are available. One question raised by these findings is the extent to which pregnant women, and women of reproductive age who may be planning pregnancy, have equivalent access to non-drug as to drug options for common mental health concerns.

## Competing interests

The authors declare they have no competing interests.

## Authors’ contributions

GEH participated in devising the analytical plan, carried our data analysis and drafted the manuscript. BM participated in devising the analytical plan, and edited the draft. Both authors read and approved the final manuscript.

## Pre-publication history

The pre-publication history for this paper can be accessed here:

http://www.biomedcentral.com/1471-2393/14/242/prepub

## Supplementary Material

Additional file 1 Generic names of all psychotropic medicines prescribed during pregnancy to women in our cohort. Click here for file

Additional file 2: Table S1 Mental health ICD-9 codes. Click here for file
