# Supplementary material for: Patterns of psychotropic medicine use in pregnancy in the United States from 2006 to 2011 among women with private insurance
Source: BMC Pregnancy Childbirth. 2014 Jul 22;14:242. doi: 10.1186/1471-2393-14-242 (PMC4223368; doi:10.1186/1471-2393-14-242)
Supplement: Additional file 1 — Generic names of all psychotropic medicines prescribed during pregnancy to women in our cohort. [file 1471-2393-14-242-S1.docx]

Additional file 1: Generic names of all psychotropic medicines prescribed during pregnancy to women in our cohort

**Antidepressants**: Amitryptiline; Bupropion; Citalopram; Clomipramine; Desipramine; Desvenlafaxine; Doxepin; Duloxetine; Escitalopram; Fluoxetine; Fluvoxamine; Hydroxytryptophan; Imipramine; Mirtazapine; Nefazodone; Nortriptyline; Paroxetine; Phenelzine; Protriptyline; Sertraline; Tranylcypromine; Trazodone; Venlafaxine; Vilazodone

**Antipsychotics**: Aripiprazole; Asenapine; Chlorpromazine; Clozapine; Fluphenazine; Haloperidol; Lithium; Loxapine; Lurasidone; Olanzapine; Paliperidone; Perphenazine; Pimozide; Prochlorperazine; Quetiapine; Thioridazine; Trifluoperazine; Ziprasidone

**Anxiolytics**: Alprazolam; Buspirone; Chloral Hydrate; Chlordiazepoxide; Clorazepate; Diazepam; Dischlorphenazone; Estazolam; Eszopiclone; Flurazepam; Lorazepam; Melatonin; Meprobamate; Methscopolamine; Midazolam; Oxazepam; Phenobarbital; Quazepam; Ramelteon; Scopolamine; Secobarbital; Temazepam; Triazolam; Zaleplon; Zolpidem

**Stimulants/ADHD medicines:** Amphetamine; Armodafinil; Atomoxetine; Dexmethylphenidate; Dextroamphetamine; Lisdexamfetamine; Methamphetamine; Methylphenidate; Modafinil
