# Supplementary material for: Patterns of psychotropic medicine use in pregnancy in the United States from 2006 to 2011 among women with private insurance
Source: BMC Pregnancy Childbirth. 2014 Jul 22;14:242. doi: 10.1186/1471-2393-14-242 (PMC4223368; doi:10.1186/1471-2393-14-242)
Supplement: Additional file 2: Table S1 — Mental health ICD-9 codes. [file 1471-2393-14-242-S2.docx]

Additional file 2: Table S1: Mental health ICD-9 codes

| Psychiatric condition | ICD-9 codes | Explanation |
| --- | --- | --- |
| Bipolar disorder | 296.0 | Bipolar I disorder, single manic episode |
|  | 296.1 | Manic disorder, recurrent episode |
|  | 296.4 | Bipolar I disorder, most recent episode (or current) manic |
|  | 296.5 | Bipolar I disorder, most recent episode or current depressed |
|  | 296.6 | Bipolar I disorder, most recent episode (or current) mixed |
|  | 296.7 | Bipolar I disorder, most recent episode (or current) unspecified) |
|  | 296.8 | Bipolar disorder, unspecified |
| Major depressive disorder | 296.2 | Major depressive disorder, single episode |
|  | 296.3 | Major depressive disorder, recurrent episode |
|  | 311 | Depressive disorder, not elsewhere classified |
| Anxiety | 300 | Anxiety, dissociative and somatoform disorders |
| Acute stress and adjustment reaction | 308 | Acute reaction to stress |
|  | 309 | Adjustment reaction |
| Schizophrenia | 295 | Schizophrenic disorders |
| ADHD | 314 | Hyperkinetic syndrome of childhood |
| Personality disorder | 301 | Personality disorders |
| Sleep | 780.5 | Sleep disturbances |
